# Identification of fibroblast activation-related genes in two acute kidney injury models

**DOI:** 10.7717/peerj.10926

**Published:** 2021-03-18

**Authors:** Weiming Deng, Xiangling Wei, Zhanwen Dong, Jinhua Zhang, Zhengyu Huang, Ning Na

**Affiliations:** Department of Kidney Transplantation, The Third Affiliated Hospital of Sun Yat-sen University, Guangzhou, Guangdong Province, China

**Keywords:** Fibroblast activation, Acute kidney injury, Bioinformatics analysis, Differentially expressed genes, Hub genes

## Abstract

**Background:**

Ischemia-reperfusion injury and drug-induced nephrotoxicity are the two most common reasons for acute kidney injury (AKI). However, little attention has been paid to early activation of fibroblasts in the progression of AKI to chronic kidney disease (CKD). The present study aimed to identify related genes and pathways on fibroblast activation in two mouse models of AKI: ischemia-reperfusion injury (IRI) model and folic acid (FA)-induced injury model.

**Methods:**

The microarray expression profiles of GSE62732 and GSE121190 were downloaded from the GEO database, and the differentially expressed genes (DEGs) was analyzed using the Limma package of R software. Principal component analysis (PCA) was also performed using R. The functional information of gene products was annotated by Gene Ontology (GO) and DAVID online database, and the pathway analysis was carried out by using the Kyoto Encyclopedia of Genes and Genomes pathway (KEGG) database. Protein-protein interactions (PPI) network was constructed by STRING and Cytoscape. Furthermore, in the Hypoxia/Reoxygenation (H/R) model, the morphological changes of cells were observed under microscope and the expression of the hub genes in NRK-49F cells were validated by qRT-PCR assays.

**Results:**

A total of 457 DEGs were identified. Among these, 215 DEGs were upregulated and 242 DEGs were downregulated in the acute injured samples compared with uninjured samples. The GO enrichment analysis indicated that these DEGs were mainly involved in transport, the oxidation-reduction process, the metabolic process, metal ion binding, hydrolase activity, and oxidoreductase activity. The KEGG analysis revealed that these DEGs were significantly enriched in the PI3K-Akt signaling pathway, protein digestion and absorption pathway, and focal adhesion pathway. The hub genes including Hnf4α, Pck1 and Timp1 were validated by the qRT-PCR assay in NRK-49F cells in the H/R model.

**Conclusions:**

Hnf4α, Pck1 and Timp-1 may play a pivotal role in the early activation of fibroblasts, providing novel therapeutic strategies for early prediction and treatment of renal fibrosis.

## Introduction

Acute kidney injury (AKI) is a renal complication that occurs in up to 20% of hospital admissions (Heung & Chawla, 2012), and part of it can eventually progress to chronic kidney disease (CKD) through interstitial fibrosis (Rodriguez-Romo et al., 2016). Both AKI and CKD represent significant health burdens and devastating conditions with high morbidity and mortality (Basile et al., 2016; Rewa & Bagshaw, 2014). Myofibroblasts (MFs) are the principal cells responsible for extracellular matrix (ECM) production. Generally, MFs do not exist in normal kidneys, while 50% of MFs in fibrotic kidneys are derive from renal resident fibroblasts (LeBleu et al., 2013). The activation of fibroblasts and excessive deposition of the ECM leads to damage of the renal parenchyma and progressive loss of renal function, which eventually progresses to end-stage renal disease (ESRD) (Eddy, 2005). Thus, renal fibrosis is the hallmark of CKD, and it has been demonstrated that the activation of fibroblasts leads to fibrosis (Grgic et al., 2012). However, the molecular mechanisms underlying fibroblast activation are not entirely understood.

Recently, some scholars have found that the activation of Sirtuin 2(SIRT2) promotes fibroblast activation and aggravates renal fibrogenesis (Ponnusamy et al., 2015). As expected, its inhibitor suppresses fibroblast activation through murine double minute2(MDM2), suggesting the SIRT2-MDM2 signaling pathway may be a therapeutic target for renal tubulointerstitial fibrosis (He et al., 2018). In the study of ischemic renal fibrosis, Cyr61 may not only inhibit the fibrotic phenotype of fibroblasts, but also inhibit the proliferation of fibroblasts through the p53/P21/Rb pathway (Liu et al., 2019). Glycogen synthase kinase 3 (GSK3) could promote renal fibrosis through TGF-β signaling, and the pharmacological inhibition of GSK3 could suppress fibroblast activation, even after the detection of AKI in patients (Singh et al., 2015). At present, most studies have proved a detrimental role of the fibroblast activation of renal fibrosis on CKD. Furthermore, the role of fibroblast activation of AKI has been largely ignored. It was reported that fibroblast activation is an extremely early event in AKI, and the blockade of early activation of fibroblasts by genetic and pharmacologic approaches aggravate renal damage and dysfunction after AKI, indicating that renal fibrosis is a complex and multistep pathological process (Zhou et al., 2019). Thus, there is an urgent need to identify novel and predictive biomarkers as therapeutic targets to interfere the progression of AKI to CKD.

The present study identified differentially expressed genes (DEGs) in kidney ischemia-reperfusion injury (IRI) and folic acid (FA)-induced injury models of C57BL/6 mice using the bioinformatics analysis of Gene Expression Omnibus (GEO) database. Then, functional enrichment analysis including Gene Ontology (GO) and KEGG, as well as protein-protein interaction (PPI) network analysis were carried out to explore the potential pathways and hub genes for further research.

## Materials and Methods

### Microarray data analysis

The GEO database (http://www.ncbi.nlm.nih.gov/geo/) was searched using ‘(Acute Kidney Injury) AND Fibroblasts’, and two datasets were selected for the present analysis. Dataset GSE62732 was based on the GPL1261 platform (Affymetrix Mouse Genome 430 2.0 Array), which included five ischemic samples and three normal samples (Bechtel et al., 2010). Dataset GSE121190 was also based on the GPL11180 platform (Affymetrix HT MG-430 PM Array Plate), which included three FA-induced samples and three uninjured kidney samples (Higashi, Aronow & Dressler, 2019). Subsequently, the data obtained from these two datasets were assessed using the R software.

### Screening of DEGs

The original data was normalized using the robust multi-array average (RMA) method. The average of multiple probes for a single gene was calculated. The linear model analysis of microarray data package (LIMMA) in R was used to identify DEGs in AKI samples compared with the non-injured renal samples. The log_2_ of the fold change (logFC) was calculated. The —logFC—>1 and corrected *P*-value of <0.05 were set as the cut-off criteria. The venn diagram and the heatmap were generated by using the online tool imageGP (http://www.ehbio.com/ImageGP/index.php/Home/Index/index.html). The volcano plot was generated using the “ggplot2” R package (https://ggplot2-book.org/).Principal component analysis (PCA) was performed to assess the similarity of gene expression patterns in the two different AKI models by using the “gmodels” R package (https://cran.r-project.org/web/packages/gmodels/index.html).

### Function and pathway enrichment analysis of DEGs

The Database for Annotation, visualization and Integrated Discovery (DAVID) online database (V6.8; http://david.abcc.ncifcrf.gov) was used to perform functional analyses namely biological process (BP), molecular function (MF), and cellular component (CC), as well as KEGG pathway analyses in May, 2020 (Huang da, Sherman & Lempicki, 2009). Terms with *P* < 0.05 were considered statistically significant.

### Construction of the protein-protein interaction (PPI) network and module analysis

The PPI network was constructed using online STRING database (v11.0; http://www.string-db.org/), which is a database of known and predicted protein interactions (Szklarczyk et al., 2011). Then, the DEGs were imported to STRING for analysis, and merely interactions with a score of >0.7 were pasted into the Cytoscape software (v3.7.2; https://cytoscape.org). Subsequently, Molecular Complex Detection (MCODE) and Cytohubba plugin of Cytoscape sofeware were used to identify potential modules and key genes in the PPI network (Chin et al., 2014). The criteria in the MCODE plugin were set, as follows: degree cut-off = 2, node score cut-off = 0.2, k-core = 2, and maximum depth = 100.

### Cell culture and the hypoxia/reoxygenation (H/R) model

NRK-49F cells were purchased from the American Type Culture Collection, and incubated in Dulbecco’s Modified Eagle’s Medium (DMEM) (Invitrogen, USA), supplemented with 10% fetal bovine serum (GIBCO) and 1% penicillin /streptomycin, under the conditions of 5% carbon dioxide (CO_2_) and 95% air at 37 °C. In order to establish the hypoxia/reoxygenation (H/R) injury model, the renal fibroblasts were deprived of serum and exposed to hypoxia (37 °C, 1% oxygen, 94% nitrogen and 5% CO_2_) for 12 h to induce hypoxic injury. Subsequently, the culture medium was refreshed, and the culture plate was reoxygenated in a regular incubator (5% CO_2_ and 95% air) for 6 h, while the control group was cultured in an ordinary cell incubator.

### Morphologic observation under inverted phase contrast microscope

NRK-49F cells were equally seeded in 60 mm culture dishes (430166, Corning, USA) at a density of 5 × 10^6^ cells/plate. The cells were treated with hypoxia for 12 h and reoxygenation for 6 h according to the method of aforementioned H/R model. Three repeated dishes were performed, and the morphology changes of cells was observed under an inverted phase contrast microscope (Olympus, Tokyo, Japan) after being washed three times with PBS. In addition, the ImageJ software (Version 1.52) was used to count cells in the captured images.

### Validation of hub genes by qRT-PCR

In the present study, qRT-PCR assay was used to validate the expression levels of 10 hub genes with the highest degree of interaction and significance in the PPI network. TRIzol reagent (Thermo Fisher Scientific, Inc.) was used to isolate total RNA from the H/R model and control group. Then, the RNA was reverse transcribed into complementary DNA according manufacturer’s instructions of the Promega kit (Promega Corp., USA). Using the ABI PRISM 7500 (Thermo Fisher Scientific, Inc.) to measure the mRNA expression levels of hub genes by qRT-PCR reactions,and *β*-actin was used as an internal reference. Three replicate holes were performed for the experiment, and the primer sequences are shown in Table 1. The relative expression was calculated using the 2^−ΔΔ*Ct*^ method, with normalization to the *β*-actin gene.

**Table 1:** Primers used for the study.

**Gene**	**Forward primer**	**Reverse primer**
α-SMA	CTATGAGCTGCCTGACGGG	GCTGTTGTAGGTGGTCTCATGG
Timp1	GGTTCCCTGGCATAATCTGA	ATGGCTGAACAGGGAAACAC
Hgd	GCCCCTACAATCTCTACGCT	TTGTGGTTGATGTGGCCTTG
Pipox	CTCTCCAGACCCTTCGGATC	GCATGGGAAAGCTTGTGACA
Slc2a2	AGTCACACCAGCACATACGA	TGGCTTTGATCCTTCCGAGT
Myc	ATCCTGTCCGTTCAAGCAGA	TGTTTCAACTGTTCTCGCCG
Spp2	AGGGACAGGTGAAGGATGTG	TTCTTCGTCGGTGGGATCTT
Vcan	GGGAAGGTTTTGTGCCAGAG	GGCTTTCCTGAGAGAGTGGT
Hao2	TGCTCTGAGAGAAGTGGTGG	CATCAGTGCCAGTTCGAACC
Hnf4α	TACTTGGTCATGGTCAGTGT	GACCCTGTGAGGGCATAAGG
Pck1	TGGGAACTCACTGCTTGGGA	AGTTATGCCCAGGATCAGCAT
*β*-actin	CGCGAGTACAACCTTCTTGC	CGTCATCCATGGCGAACTGG

### Statistical analysis

GraphPad Prism 8.0 (GraphPad Software, Inc., San Diego, CA, USA) was used for the statistical analysis of all data. R software version 3.5.0. was used for analysis. The qRT-PCR results were presented as mean ± standard deviation. The differences of cell count in captured pictures and the qRT-PCR results were analyzed by Student’s *t*-test. *P* < 0.05 was considered statistically significant.

## Results

### DEGs screening

**Figure 1:**
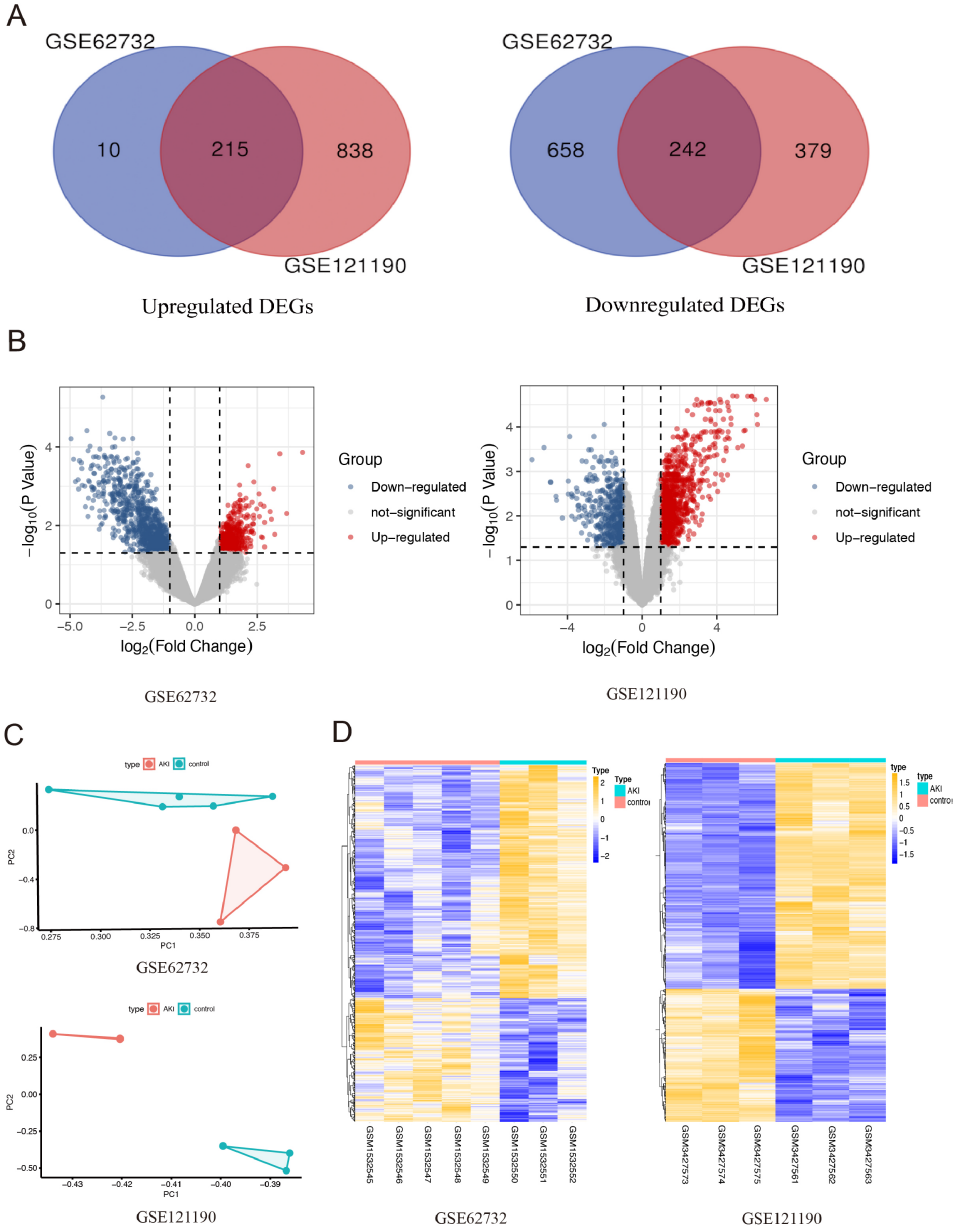
The screening for differentially expressed genes in the two AKI models. (A) The Venn diagram presents the common 215 co-upregulated and 242 co-downregulated DEGs in the GSE62352 and GSE121190 datasets. (B) The volcano plot shows distribution of DEGs of the GSE62352 and GSE121190 datasets. (C) Principal component analysis (PCA) was performed to assess the transcription profile between GSE62352 and GSE121190 datasets, the results showed that there were significant differences between the two subgroups. (D) The heatmap shows the expression level of the upregulated and downregulated DEGs extracted from GSE62352 and GSE121190 datasets.

After integrated analysis of the two datasets (GSE62732 and GSE121190) between acute injured kidney and normal samples, 457 DEGs (—log2 FC— >1 and FDR <0.05, Figures1) were screened, which included 215 upregulated genes and 242 downregulated genes. Venn diagram and volcano plots (Figs. 1A and 1B) were generated to present the correlation between DEGs. Principal component analysis (PCA) was performed to identify similar gene expression patterns in samples between two subgroups. The results showed that there were significant distinctions between the two subgroups Fig. 1C). A heatmap was constructed to visually present the expression change of the upregulated and downregulated DEGs in the two datasets, and reveal the distribution of the gene expression data of each subset (Fig. 1D).

### The GO and KEGG pathway enrichment analysis

**Figure 2:**
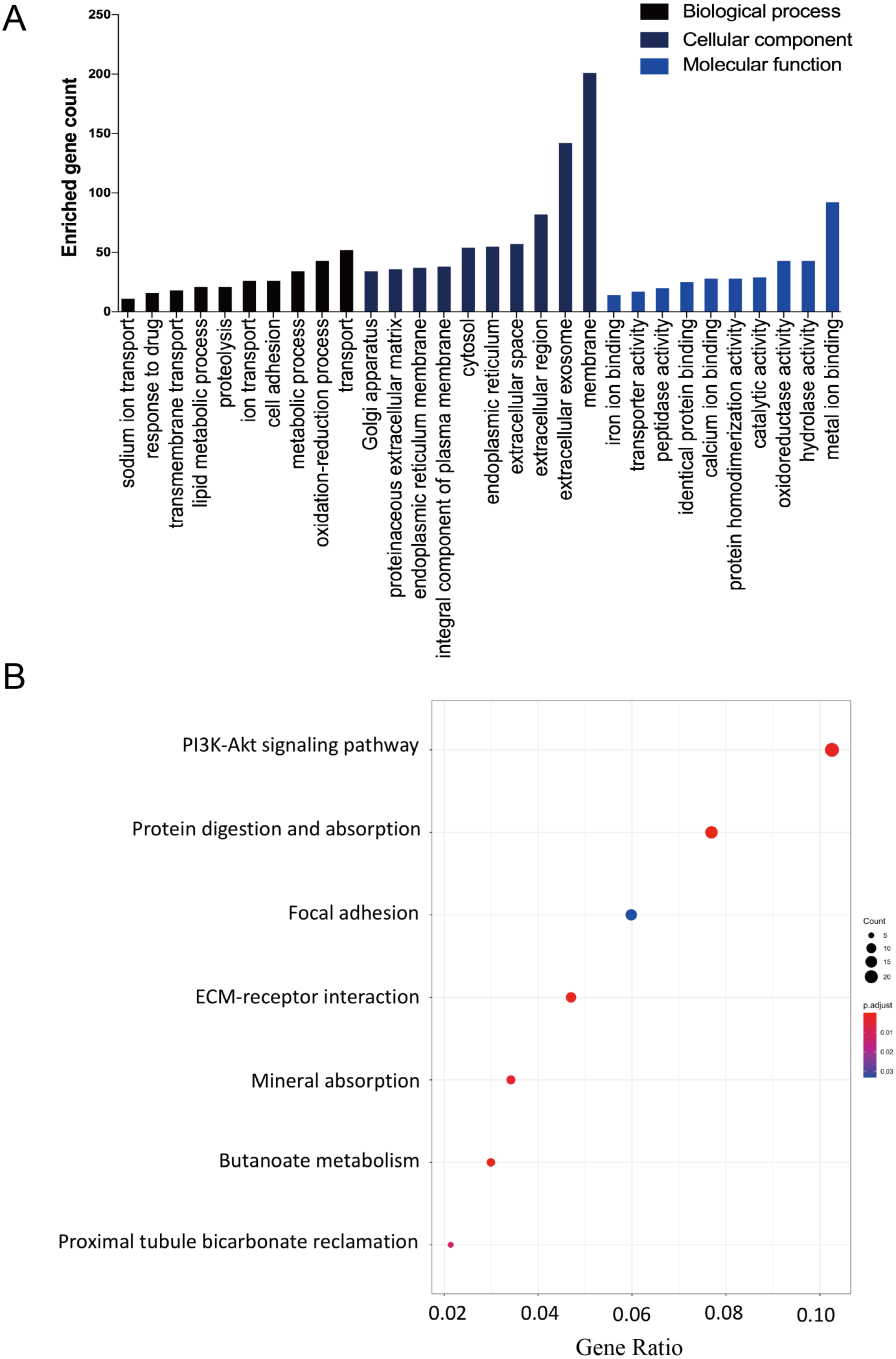
The GO functional and KEGG pathway enrichment analyses of the two AKI models. (A) The column diagram shows the top 10 enrichment scores in terms of biological processes, molecular functions, and cellular components of all DEGs. (B) The bubble plot shows the top seven KEGG pathways of all DEGs.

The DAVID database was used to determine potential function of DEGs and identify the overrepresented GO categories in the biological process of these 457 DEGs. The top 10 GO terms from the biological process (BP), cellular component (CC) and molecular function (MF) domains in the co-expressed DEGs were ranked according to enriched gene count and *P*-value (Fig. 2A). In the BP domain, the most meaningful enriched GO terms primarily focused on transport (GO:0006810), the oxidation–reduction process (GO:0055114), and the metabolic process (GO:0008152). The most enriched GO CC terms were correlated to the membrane (GO:0016020), extracellular exosome (GO:0070062), and extracellular region (GO:0005576). For the MF terms, the represented terms were metal ion binding (GO:0046872), hydrolase activity (GO:0016787), and oxidoreductase activity (GO:0016491).

**Figure 3:**
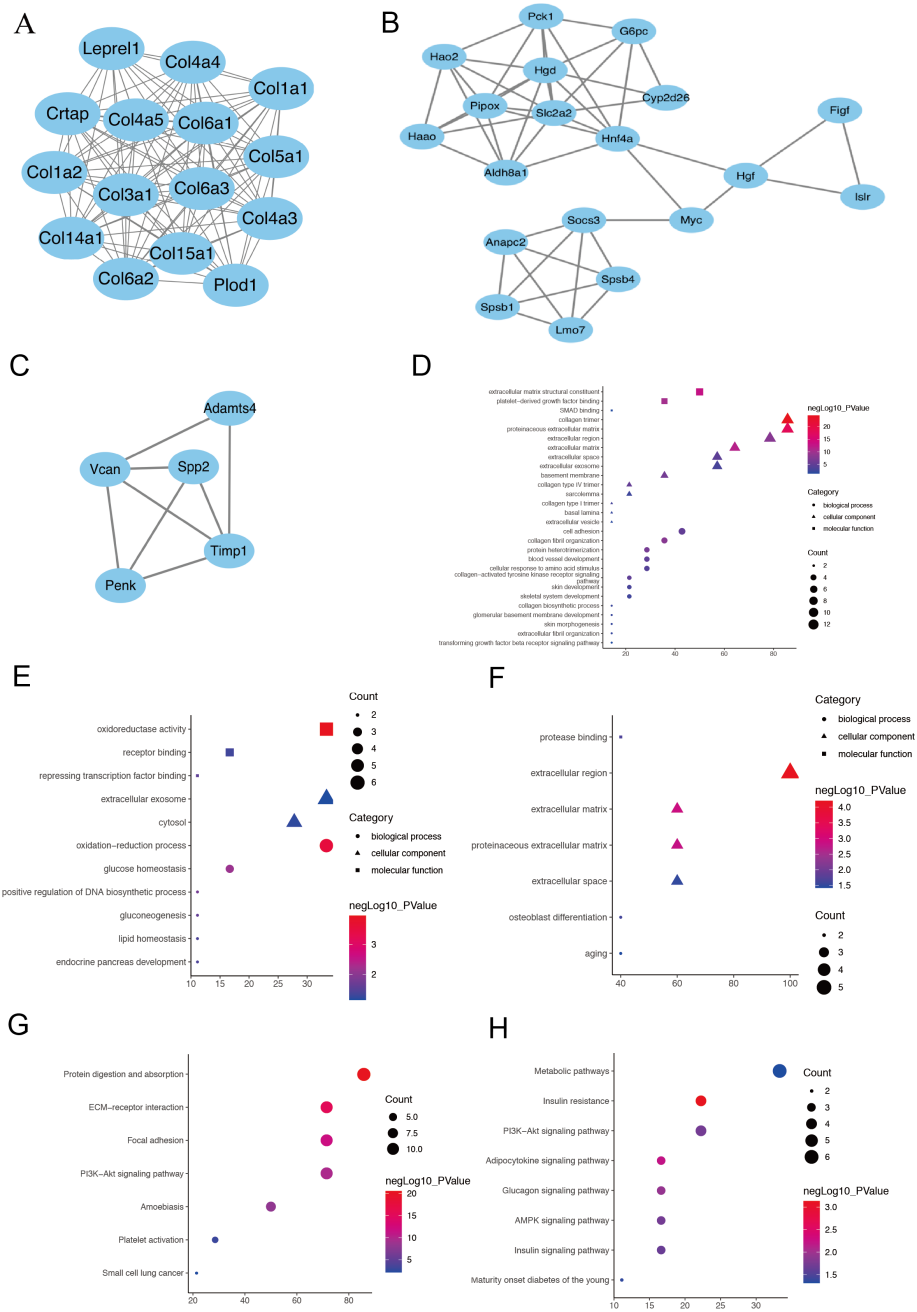
The PPI network of three prominent modules. (A, B and C) Combined with the analysis of Cytoscape and MCODE, 10 hub genes with the highest degree score were screened out from the three most prominent modules.Module 1 shows a large number of ECM-related proteins interconnected, suggesting that fibroblasts in the two AKI models have been activated and differentiated into myofibroblasts (A). (Hgd, Pipox, Slc2a2, Myc, Hao2, Hnf4α and Pck1) in module 2(B) and (Timp1, Spp2 and Vcan) in module 3(C). The module genes from the PPI analysis were identified using the plugin MCODE, and the GO as well as KEGG analysis of these three most important modules were performed using the DAVID database (D–H).

The KEGG pathway enrichment analysis results were listed and ranked by enriched gene count and *P*-value. Overall, the top seven pathways were selected from the 269 pathways correlated to fibroblast activation in the IRI and FA-induced kidney injury models (Fig. 2B). The common pathways in both sets were the PI3K-Akt signaling pathway (mmu04151), protein digestion and absorption (mmu04974), and focal adhesion (mmu04510).

### PPI network and module analysis

The PPI network was constructed using the STRING database and Cytoscape software to investigate the interactions, and acquire the hub genes for fibroblast activation-associated DEGs in AKI. Then, 393 nodes and 1,582 interactions, including 10 leading genes defined as hub genes, were selected for inclusion in the PPI network, according to the criteria for the ‘combined score of >0.7’. Module 1 shows a large number of ECM-related proteins interconnected, suggesting that fibroblasts in the two acute kidney injury models have been activated and differentiated into myofibroblasts (Fig. 3A). In addition to ECM-related genes, ten hub genes were selected in module 2 (Fig. 3B) and module 3 (Fig. 3C) by the highest degree of interaction and the most prominent modules: Timp1, Hgd, Pipox, Slc2a2, Myc, Spp2, Vcan, Hao2, Hnf4α and Pck1 (Supplementary sheet 1). Next, the module genes from the PPI analysis were identified using the plugin MCODE, and the functional annotation and pathway analysis of these two most important modules were performed using the DAVID database. In terms of BP in GO analysis, module 1 primarily focused on cell adhesion and collagen fibril organization (Fig. 3D), module 2 focused on oxidation–reduction process and glucose homeostasis (Fig. 3E), module 3 was mainly associated with osteoblast differentiation and senescence (Fig. 3F). For the KEGG pathway enrichment analysis, the represented terms of module1 were protein digestion and absorption pathway and ECM-receptor interaction pathway (Fig. 3G), while mode 2 was mainly enriched in insulin resistance pathway and adipocytokine signaling pathway (Fig. 3H). Module 3 did not enrich meaningful pathway because of its small number of genes.

### Observation of cell morphology

**Figure 4:**
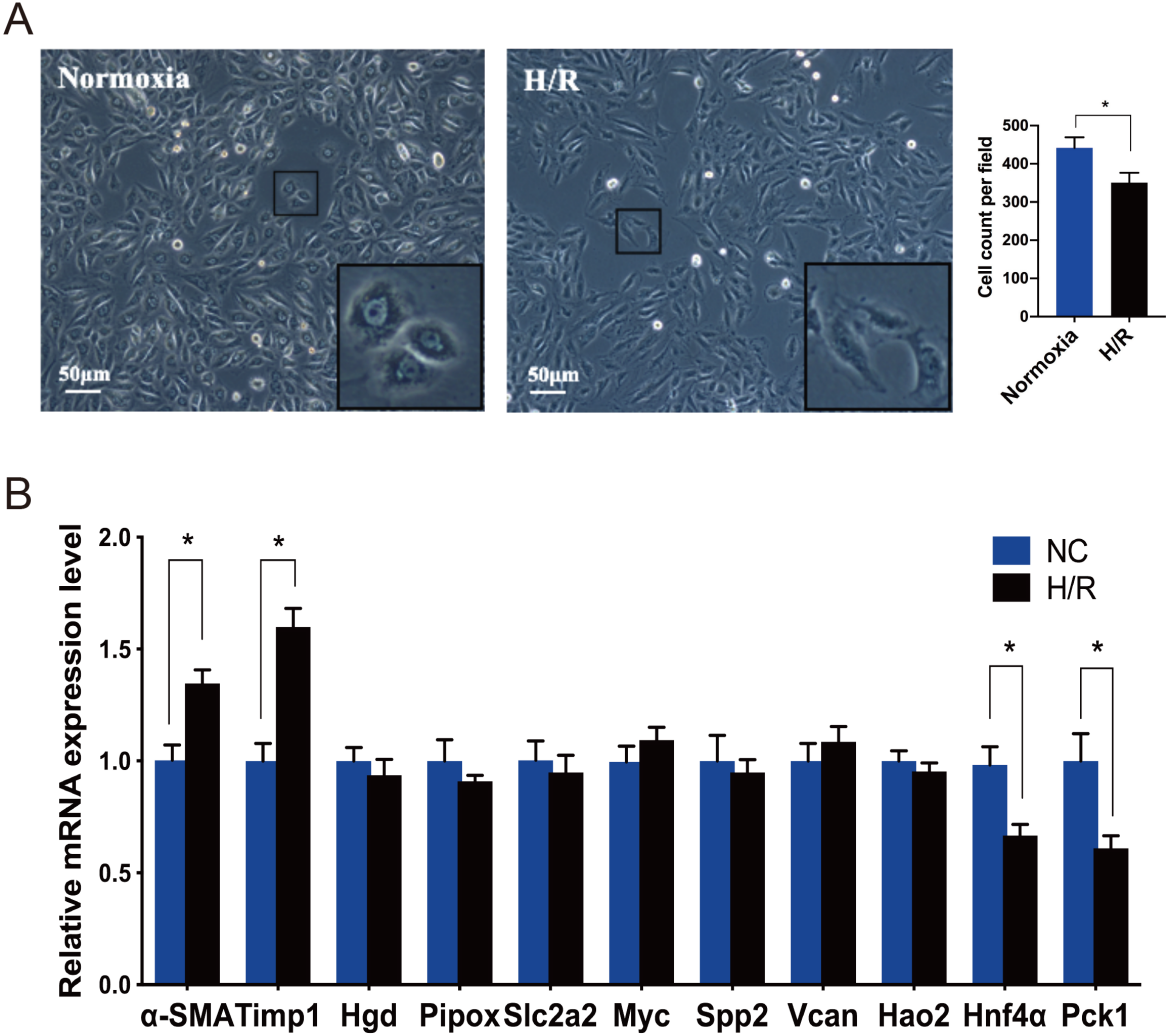
Morphological changes of NRK-49F cells and validation of hub genes by qRT-PCR. (A) Under inverted phase contrast microscope (x100), the morphology of NRK-49F cells in the control group was fusiform or spindle-shaped. After hypoxia for 12 h and reoxygenation for 6 h, the cell density decreased slightly. Moreover, the cells became longer and showed the characteristics of muscle fiber cells. (B) The qRT-PCR shows that Hnf4α and Pck-1 were downregulated and Timp-1 was upregulated in the H/R model. There was no significant difference in the expression level of the other seven genes between the H/R model and control group. *β*-actin was used as an internal control. The data were expressed as mean ± standard deviation, and * *P* < 0.05*vs.* the NC (negative control) group (*n* = 3 per group).

Under an inverted phase contrast microscope (X100), we observed the cellular morphology of NRK-49F cells exposed to normoxia and H/R conditions (Fig. 4A). The cells grown under normoxic conditions showed normal fusiform or spindle-shaped, while the cells induced by H/R were longer and the number of cells decreased. Compared with the control group, the growth rate of cells reduced slightly after H/R injury, the fibroblasts were then activated and showed the characteristics of muscle fiber cells.

### Verification of the top 10 fibroblast activation-associated DEGs through the analysis of rat renal fibroblasts

Every DEG analyzed with Cytoscape has an interactional degree with others. The 10 hub genes (Timp1, Hgd, Pipox, Slc2a2, Myc, Spp2, Vcan, Hao2, Hnf4α and Pck1), which had the highest interactional degree among all DEGs, were selected as the most prominent fibroblast activation-associated DEGs. The qRT-PCR analysis was performed to verify the expression levels of these hub genes. α-SMA is a marker of activation and differentiation of fibroblasts into myofibroblasts. As identified by the above analyses in the IRI and FA-induced injury model, Timp1 was an upregulated DEG, and the expression level was confirmed to be elevated in the H/R model, while Hnf4α and Pck1 were the downregulated DEGs, and the expression levels decreased, when compared to normal cultured fibroblasts. The difference in expression level of the other seven genes was not statistically significant between the H/R model and control group (Fig. 4B).

## Discussion

Tubulointerstitial fibrosis is the common endpoint of virtually all progressive renal diseases, regardless of the primary cause (Boor, Ostendorf & Floege, 2010). The transformation of fibroblasts to myofibroblasts is the core event in the development of renal fibrosis (Zeisberg & Neilson, 2010). Early diagnosis and intervention are particularly essential to delay the further deterioration of renal function. Therefore, it is necessary to detect the biomarkers for the early activation of fibroblasts. To our knowledge, little attention has been given to the mechanism of fibroblast activation in AKI. In order to explore the related genes that may play an important role in this process, the gene expression profiles of fibroblasts in two different AKI models of IRI and FA-induced injury were detected by bioinformatics analysis. A total of 454 differentially expressed mRNAs were identified, which included 215 upregulated mRNAs and 242 downregulated mRNAs.

In the GO analysis, it was found that the most enriched BP and MF terms were correlated to intracellular energy metabolism and redox reactions. Recently, emerging evidence has confirmed that the energy metabolism in the progression of renal fibrosis changes from mitochondrial oxidative phosphorylation to glycolysis (Lan et al., 2016; Liu et al., 2017). The results of the KEGG pathway enrichment indicated that the PI3K-Akt signaling pathway was the most significantly enriched pathway between acute injured kidney and non-injured kidney samples. A previous study demonstrated that LPS activates the PI3K-Akt-mTOR/PFKFB3 pathway, and promotes the collagen synthesis of lung fibroblasts through aerobic glycolysis to aggravate pulmonary fibrosis (Hu et al., 2020). Furthermore, *in vivo* and *in vitro* evidences have suggested that DHA and Nrf2 may attenuate renal fibrosis through the regulation of fibroblast proliferation and differentiation by inhibiting the PI3K/AKT pathway, and both of these have been used as therapeutic antifibrotic targets for the treatment of renal fibrosis (Wang et al., 2019; Zhang et al., 2019). The PPI network was constructed using the STRING online database and Cytoscape software. A total of 10 hub genes were identified according to the degree of connectivity. Then, qRT-PCR assay was performed for the H/R model and normal cultured primary fibroblasts. Then, three hub genes (Hnf4α, Pck1 and Timp-1), which were closely correlated to fibroblast activation, were filtered out in AKI.

Hnf4α is a cell-specific transcription factor in the nuclear hormone receptor superfamily (Grigo et al., 2008). This is required for homeostasis and metabolism. Furthermore, this is highly expressed in the liver, kidneys and intestines, but is downregulated in the pancreas, stomach and epididymis in adult vertebrates (Odom et al., 2004; Tanaka et al., 2006).In the study of hepatic fibrosis, it was found that the progression of hepatic fibrosis was correlated to the decreased expression of Hnf4α. The overexpression of Hnf4α could promote the expression of E-cadherin, and significantly inhibit the expression of some profibrogenic markers, such as α-SMA, Timp1 and other interstitial markers. Furthermore, inducing the mesenchymal-epithelial transition (MET) process deactivates myofibroblasts and ameliorates the ECM deposition (Yue et al., 2010). At present, no direct evidence has confirmed whether Hnf4α can block renal fibrosis. However, some scholars have proven that the combined expression of four transcription factors, including Hnf4α, has the ability to convert mouse and human fibroblasts to induced-renal tubular epithelial cells (Kaminski et al., 2016). During kidney development, it has been confirmed that Hnf4α is involved in the development of metanephric mesenchyme (MM) into nephrons through MET. In fibroblasts with a forced expression of Hnf4α protein, it was observed that Hnf4α induced the increased expression of epithelial cells (Krt8 and CdH1), and decreased the interstitial gene expression (Acta1 and Vim) (Kanazawa et al., 2011). Meanwhile, the gene expression profiling of unilateral ureteral obstruction (UUO) revealed that Hnf4α might play an important role in early gene expression events related to kidney injury and fibrosis caused by an obstruction (Wu & Brooks, 2012). Therefore, the decreased expression of Hnf4α after the early activation of fibroblasts in AKI may aggravate renal fibrosis by promoting EMT.

Pck1 is a member of the phosphoenolpyruvate carboxykinase family, which is located in the cytoplasm, and is also known as PEPCK-C. This catalyzes the conversion of oxaloacetate to phosphoenolpyruvate, which is the rate-limiting enzyme of gluconeogenesis (Tontonoz et al., 1995). The kidney is one of the most important sites of gluconeogenesis in humans, and the downregulation of key gluconeogenic enzymes, including Pck1, would deleteriously affect RCC cancer cell proliferation and survival (Khan et al., 2015). From the mechanism of cellular energy metabolism, it was found that the main feature of fibroblast activation in the process of renal fibrosis was the transition from oxidative phosphorylation to glycolysis, and that inhibiting glycolysis could suppress fibroblast activation and reduce renal fibrosis in the UUO model (Ding et al., 2017; Wei et al., 2019). Gluconeogenesis and glycolysis are two opposite pathways. When the level of intracellular energy decreases, this is conducive to glycolysis, but not to gluconeogenesis, and the factors that can reduce the level of gluconeogenesis would in turn improve the energy supply of glycolysis. Aerobic glycolysis has been considered as the dominating metabolic pathway of renal tubular epithelial cells in the progression of AKI. Glycolysis inhibitors can suppress fibroblast activation after FA-induced injury through the early inhibition of lactate production in renal tubules (Shen et al., 2020). Recent studies have found that the transcription level of Pck1 is downregulated in the acute renal injury stage of FA-induced fibrosis, suggesting that this participates in mitochondrial energy metabolism, and promotes the progression of renal interstitial fibrosis (Shen et al., 2018). Compared with the detection of total RNA in mouse kidneys, the transcript level of Pck1 in fibroblast mRNA alone would be more meaningful when determining whether this can be used as a precise marker of renal fibrosis.

Timp1 is a member of the tissue inhibitors of metalloproteinases (TIMPS), which are endogenous protein regulators of the family of matrix metalloproteinases (MMPs) (Murphy, 2011). Except for inhibiting matrix degradation, Timp1 is involved in inflammation, and cell proliferation and apoptosis (Peng et al., 2011). In the study of sepsis-associated acute kidney injury (SA-AKI), septic patients had higher serum TIMP-1 levels which could serve as a potential diagnostic biomarker of SA-AKI (Bojic et al., 2015). As a recognized profibrogenic-marker, Timp1 plays a variety of roles in the progression of fibrotic diseases. In the UUO model, the overexpression of Timp1 deteriorates renal interstitial fibrosis through the inflammatory pathway induced by upregulating ICAM-1l (Cai et al., 2008). It has been identified that Timp1 stimulates fibroblasts activation and proliferation via the interaction of CD63/integrin *β*1, and the activation of the intracellular ERK pathway in the development of lung fibrosis (Dong & Ma, 2017). In addition, hypoxia also increased the expression of Timp1 in mouse fibroblast AKR-2B cells, and induced a phenotypic switch of fibroblasts to myofibroblasts through the MMP-2/TIMP mediated pathway (Misra et al., 2010). The expression level of Timp1 varies in different stages and cells of fibrosis. It has been found that Timp1 is active in the early stage of fibrosis in the UUO model, and initial Timp1 mRNA was transcribed by unidentified interstitial cells and few ED1+ macrophages, but this was mainly transcribed by α-SMA+ myofibroblasts until the later stage of post-ligation (Duymelinck et al., 2000). Nevertheless it is noteworthy that the elimination of Timp1 alone did not block the progression of renal interstitial fibrosis, which might be due to the compensatory effect of other protease inhibitors, such as TIMP-2, TIMP-3, or PAI-1 (Kim et al., 2001). Different from previous studies that focused on chronic fibrosis models, it was found that the expression of Timp1 increased in fibroblasts in two different AKI models, suggesting that the early fibroblast activation that led to renal fibrosis is an early event in the process of AKI transition to CKD.

## Conclusions

In summary, a comprehensive bioinformatics analysis of the possible DEGs and pathways involved in two different AKI models was conducted. DEGs, such as Hnf4α, Pck1 and Timp-1, as well as the PI3K-Akt signaling pathway, protein digestion and absorption pathway, focal adhesion pathway and gluconeogenesis/glycolysis pathway, may play a pivotal role in the early activation of fibroblasts. The findings of the present study may facilitate our understanding the different roles of fibroblast activation in AKI.

However, the present study also has some limitations. First, the sample size of the microarray analysis was small, which may cause a potential false positive rate. Second, in vivo experimental verifications were absent from the present study. Therefore, in-depth experiments with a large sample size should be considered to corroborate these discoveries.

##  Supplemental Information

10.7717/peerj.10926/supp-1 Supplemental Information 1 GO enrichment

10.7717/peerj.10926/supp-2 Supplemental Information 2 String interactions

10.7717/peerj.10926/supp-3 Supplemental Information 3 GSE62732 unregulated DEGs

10.7717/peerj.10926/supp-4 Supplemental Information 4 GSE62732 downregulated DEGs

10.7717/peerj.10926/supp-5 Supplemental Information 5 GSE121190 upregulated DEGs

10.7717/peerj.10926/supp-6 Supplemental Information 6 GSE121190 downregulated DEGs

10.7717/peerj.10926/supp-7 Supplemental Information 7 GSE121190 all DEGs

10.7717/peerj.10926/supp-8 Supplemental Information 8 GSE62732 all DEGs

10.7717/peerj.10926/supp-9 Supplemental Information 9 cytoscape Module1

10.7717/peerj.10926/supp-10 Supplemental Information 10 cytoscape Module 2

10.7717/peerj.10926/supp-11 Supplemental Information 11 Cytoscape Module 3

## Additional Information and Declarations

### Data Availability

The following information was supplied regarding data availability: Data are available at NCBI GEO: GSE62732, GSE121190.
